# Oxidative Stress and the Induction of Cyclooxygenase Enzymes and Apoptosis in the Murine Placenta

**DOI:** 10.1016/j.placenta.2006.12.001

**Published:** 2007-07

**Authors:** C. Burdon, C. Mann, T. Cindrova-Davies, A.C. Ferguson-Smith, G.J. Burton

**Affiliations:** Department of Physiology, Development and Neuroscience, University of Cambridge, Cambridge CB2 3DY, UK

**Keywords:** Oxidative stress, Cyclooxygenases, Apoptosis, Murine placenta

## Abstract

Placental oxidative stress has been implicated in many complications of human pregnancy, including preterm delivery and preeclampsia. It is now appreciated that reactive oxygen species can induce a spectrum of changes, ranging from homeostatic induction of enzymes to apoptotic cell death. Little is known regarding the occurrence of placental oxidative stress in other species. We investigated markers of oxidative stress in the labyrinthine (LZ) and junctional (JZ) zones of the murine placenta across gestational age, and correlated these with expression of the cyclooxygenase enzymes COX-1 and COX-2, and apoptosis. We tested a causal link between the two by subjecting placental explants to hypoxia-reoxygenation (H/R) in vitro, a known stimulus for generation of oxidative stress. Western blotting demonstrated significant increases in the concentrations of hydroxynonenal (HNE), COX-1 and COX-2 with gestational age. Dual-labelling demonstrated co-localisation of HNE, and COX-1 and COX-2 within the trophoblast of the LZ, and glycogen cells of the JZ. An apoptotic index based on TUNEL-positivity demonstrated an increase with gestational age, and dual-labelling showed co-localisation of TUNEL labelling with HNE and active caspase-3 within the trophoblast of the LZ. H/R significantly increased oxidative stress, induction of COX-1 and COX-2, and the apoptotic index. Co-localisation demonstrated the increases in COX to be within the trophoblast of the LZ, and in particular the glycogen cells of the JZ. Apoptosis was restricted to the LZ. We speculate that the induction of COX enzymes is a physiological response to oxidative stress, and may play a role in initiating or augmenting parturition. Generation of oxidative stress may also play a role in influencing the growth trajectory of the placenta, and its component cell types. The mouse may provide an experimental genetic model in which to investigate these phenomena.

## 1 Introduction

Oxidative stress of the placenta has been implicated in the pathogenesis of many complications of human pregnancy, including miscarriage, preeclampsia, and preterm labour [1–3]. These complications all share the common predisposing feature of reduced trophoblast invasion and incomplete conversion of the uterine spiral arteries [4,5]. Therefore, it is generally held that malperfusion of the placenta leads to increased oxidative stress, resulting in placental dysfunction. In the case of preeclampsia it is thought that the stress induces the release of a cocktail of factors, including pro-inflammatory cytokines, anti-angiogenic factors and apoptotic debris [6], into the maternal circulation that causes activation of the peripheral endothelial cells. Placental inflammatory lesions have also been associated with premature onset of labour [7].

By contrast, there are few data relating to placental oxidative stress available for other species. The mouse is potentially a powerful model in which to study the mechanisms of placental and obstetrical pathologies, and is the best-studied mammalian experimental genetic model system. The definitive murine placenta is a discoid haemochorial organ as in the human, and there are three anatomically and physiologically distinct regions: the labyrinth zone (LZ), junctional zone (JZ) and decidua basalis (DB) [8]. The labyrinth zone is the principal area of exchange, and consists of a meshwork of maternal blood spaces lined by trophoblast. The trophoblast comprises three layers; an outer layer (I) composed of cytotrophoblast cells, and two layers (II & III) of syncytiotrophoblast [9]. Deep to the syncytiotrophoblast are the fetal capillaries, embedded in a small amount of connective tissue. The junctional zone, interposed between the labyrinth and the decidua basalis, is of unknown function, but contains spongiotrophoblast cells and trophoblastic glycogen cells (GC) [8].

The decidua basalis is composed principally of maternal uterine tissues, and contains maternal arteries and veins that are continuous with the arterial and venous channels traversing the JZ. Glycogen cells migrate into the DB and surround the maternal arteries that supply the placenta late in gestation. The mechanisms of myometrial stimulation and activation appear to parallel those of the human [10]. For example, prostaglandins (PGs) are central components of labour in both humans and mice [11]. There are fundamental similarities in the changes occurring prior to labour, including an increase in myometrial contractile activity, increased coupling of myometrial cells through the formation of gap junctions, an increase in PG receptors in the myometrium, and enhanced sensitivity to oxytocin at term. There is also a rise in intracellular calcium resulting in an increase in myosin light chain phosphorylation.

In this study we determined the degree of oxidative stress present in the murine placenta at different gestational ages in normal pregnancies. We also tested whether there was a temporal and spatial association between oxidative stress and two markers of placental function, one physiological and one pathological. These were the induction of cyclooxygenase (COX) enzymes and apoptosis respectively.

Evidence from other systems points to potential links between oxidative stress and COX induction, resulting in the production of PGs [12–16]. PGs are derived from arachidonic acid (AA) through the actions of the COX enzymes, which are rate-limiting. There are two known isoforms of COX, COX-1 and COX-2, which share similar structures but differ in their function. COX-1 is believed to play a constitutive “housekeeping” role, whereas COX-2 is considered to be an inducible isoform. However, these contrasting roles have not been proven in the mouse [17]. In mice induction of COX-2 by administration of lipopolysaccharide leads to preterm labour, which can be blocked using the specific COX-2 inhibitor SC-236 [18].

Apoptosis has been described within the LZ of the normal mouse placenta, and is most frequent towards term and in post-mature placentas [19]. The stimulus for this is not known, but oxidative stress is a potent inducer of trophoblast apoptosis in the human placenta [20].

## 2 Materials and methods

### 2.1 Tissue collection

Placentas were collected from C57BL/6J inbred mice, and all experiments were carried out in accordance with the UK Government Home Office licensing procedures. Stages E14, E16, E18, and E19 were studied, where E1 of gestation was the morning when a copulation plug was found.

### 2.2 Western blotting

Placentas from three different animals at each gestational age were homogenized in ice-cold sample buffer, and centrifuged at 15,000 rpm. Protein concentration within the supernatant was determined using a colorimetric assay (Bio-Rad Bradford protein assay). An equal amount of protein from each sample (30 μg) was subjected to electrophoresis in a 10% SDS-polyacrylamide gel under reducing conditions and transferred onto nitrocellulose membrane by a semidry transfer machine. Membranes were incubated in a blocking solution (TBS containing 5% powdered non-fat milk and 0.01% Tween-20) at room temperature for 1 h, and then in the same solution containing primary antibody (Table 1) at 4 °C overnight. After washes in TBS-T, membranes were incubated with the horseradish peroxidase secondary antibody at room temperature for 1 h. The immunoreactive bands were visualized by enhanced chemiluminescence with the ECL system (Amersham Biosciences) according to the manufacturer instructions. The levels of protein expression were quantified densitometrically and normalised against their respective expressions of β-actin. Prestained protein markers were used as molecular weight standards for each analysis.

### 2.3 Immunohistochemistry

At each gestational age three placentas were fixed by immersion in 4% paraformaldehyde overnight, dehydrated and embedded in paraffin wax. Sections (6 μm thick) were dewaxed and immunostained according to our standard protocol [21]. When necessary, antigen retrieval was performed in a pressure cooker for 2 min in 0.01 M Citrate buffer at pH 6. Primary antibodies (Table 1), diluted in goat serum, were applied overnight in a humidified chamber at 4 °C. Binding was detected using Vectastain Elite ABC kits (Vector Laboratories) and SigmaFast DAB (Sigma), according to the manufacturers’ instructions. Sections were then lightly counterstained with haematoxylin. Negative controls were performed by omitting incubation with the primary antibody. For each antibody slides from all the gestational ages were prepared and immunostained in the same batch, ensuring identical conditions for comparisons.

In order to localise expression of the antigens to specific cell types staining intensity was graded visually from 0–3, where 0 represented the negative control. Three people scored the sections independently, blinded to the gestational age or experimental protocol, and the mean value was taken.

### 2.4 Immunofluorescent dual-labelling

Dual immunofluorescent labelling was performed in order to test for co-localisation of markers of oxidative stress and COX expression in individual cells. Sections were dewaxed, permeabilised in TBS containing Triton X-100 (0.1%) and Tween 20 (0.1%) (TBS-TT) for 30–60 min, and blocked in 5% bovine serum albumin for 20 min at room temperature. Primary antibodies diluted in TBS-TT were applied overnight at 4 °C. Negative control sections had primary antibodies omitted. After three 10-min washes in TBS-TT, sections were incubated for 1 h at room temperature with a mixture of fluorescent secondary antibodies, containing anti-goat Alexa 488 and anti-rabbit Alexa 568 (both used 1/100; from Molecular Probes) in TBS-TT. Sections were washed in TBS-TT as before and then twice in distilled water for 5 min and subsequently mounted in Vectashield mounting medium containing DAPI (Vector, UK). Images were captured using a Leica confocal microscope (LeicaTCS-NT, Leica Instruments GmbH).

Co-localisation was identified in overlay images by a yellow signal caused by the combination of the green signal (COX-1/COX-2) and the red signal (HNE).

### 2.5 TUNEL assay

Staining was performed using In-Situ Cell Death Detection kit-Texas Red (Roche Applied Science) according to the manufacturer's instructions. Conditions were adjusted so that the staining pattern matched morphological assessments of apoptosis based on examination of resin-embedded samples. Once the protocol had been optimised an apoptotic index was calculated as follows:

Apoptotic Index(AI:%)=Number of TUNEL stained nuclei/Number of DAPI stained nuclei×100.

### 2.6 Dual-fluorescence TUNEL assay & Active Caspase-3, and HNE co-localisation

Staining was performed using In-Situ Cell Death Detection kit-Texas Red (Roche Applied Science) according to the manufacturer's instructions, and using an Anti-Active Caspase-3 antibody (Promega) at 1:50, and Anti-HNE as previously at 1:200. Samples were also DAPI stained to intercalate the DNA and fluoresce cell nuclei. For negative controls, omission of primary antibody and TdT enzyme were performed separately. For positive controls, post-lactational mouse mammary glands were used. For confocal microscopy, all samples were analysed during one session to avoid bias, and multiple (usually 5) fields of view per labyrinth were saved for further analysis.

### 2.7 Ex-vivo culture experiment

In order to induce oxidative stress placental samples were subjected to hypoxia-reoxygenation (H/R) in vitro [22]. Placentas were taken from 3 animals at gestational age E18, halved on an ice block and immersed in ice-cold PBS prior to culture. Three samples, each 5–10 mm^3^, from each placenta were incubated in culture medium (TCS large vessel endothelial cell basal medium (TCS CellWorks, Milton Keynes, UK)), which had been maintained for several hours in the specified gas composition at 37 °C. Data for intraplacental oxygen concentrations are not yet available for the mouse, and so estimates were made on the basis of results for another small mammal, the guinea-pig. The umbilical venous pO_2_ towards term is 29.5 mmHg, which is close to that for fetal primates and ungulates [23]. Therefore, for normoxia a gas mix of 5% O_2_, 90% N_2_, 5% CO_2_ was used, whereas for hypoxia it was 1% O_2_, 94% N_2_, 5% CO_2_. Gas composition was monitored and controlled throughout using Biospherix (Redfield, NY) Pro-Ox and Pro-CO2 probes. Control samples were cultured for 4 h in normoxia, whereas samples subjected to H/R experienced 1 h of hypoxia followed by 3 h of normoxia. Following culture the tissues were either frozen or fixed for Western blotting and immunohistochemistry respectively.

### 2.8 Statistical analyses

Statistical significance was calculated by using one-way analysis of variance (ANOVA). Differences between groups were assessed using Fisher's Least Significant Difference Test. Statistical significance was assumed at a *P*-value <0.05.

## 3 Results

### 3.1 Markers of oxidative stress

**Fig. 1:**
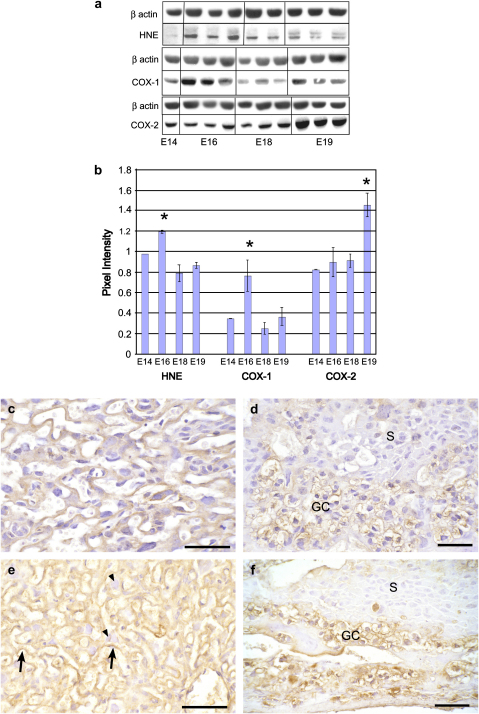
a) Representative Western blots for HNE, COX-1 and COX-2 at the different gestational stages; and b) quantification following normalisation for ß actin. Mean ± SE for placentas from 3 different litters at each gestational age. * indicates unique groups based on Fisher's protected LSD test. c–f) photomicrographs of IHC for HNE. Low levels of immunoreactivity were observed at E14 in the syncytiotrophoblast and cytotrophoblast cells in the LZ (c); and in the GCs of the JZ (d). At E18 the cytotrophoblast cells (arrowheads) still displayed relatively weak immunoreactivity, whereas strong staining was present in the syncytiotrophoblast (arrowed) within the LZ (e); and glycogen cells (GC) in the JZ (f). The spongiotrophoblast (S) cells displayed low levels of immunoreactivity at both gestational ages. Scale bars: 50 μm.

Hydroxynonenal (HNE) is a marker of lipid peroxidation. Western blotting revealed significant changes across gestational age (*P* = 0.003), with low concentrations of HNE at E14, a significant increase at E16, and a subsequent decline by E18 (Fig. 1a,b).

Scoring based on IHC revealed different intensities of immunoreactivity in the different cell populations, and different temporal patterns. At E14 immunoreactivity was low in all cell types (Fig. 1c,d). At E16 there was moderate staining (scored 2) in the syncytiotrophoblast of the LZ, which peaked (3) at E18 and declined to 1 by E19 (Fig. 1e). The cytotrophoblast cells of the LZ stained uniformly at level 1 throughout, and no immunoreactivity was observed in the fetal endothelial cells. In the JZ strong staining was observed in the GCs at E16 (2.5) and E18 (3) (Fig. 1f), and this was maintained at 2.5 at E19. The spongiotrophoblast cells were less immunoreactive, scoring only 0.5 throughout gestation, and no immunoreactivity was observed in the giant cells or the decidua.

Nitrotyrosine (NT) staining indicates the formation of the prooxidant peroxynitrite, and of an imbalance in the production of the superoxide ions and nitric oxide. Immunostaining revealed a similar pattern to HNE, with almost undetectable staining in early samples and significant increases from day 16 onwards (data not shown).

Therefore, oxidative stress increases during gestation in the murine placenta, specifically in syncytiotrophoblast and glycogen cells.

### 3.2 COX-1

Western blotting revealed significant changes in concentrations of COX-1 across gestational age (*P* = 0.013). There was an increase from E14 to E16, followed by a significant decrease by E18 (Fig. 1a,b).

**Fig. 2:**
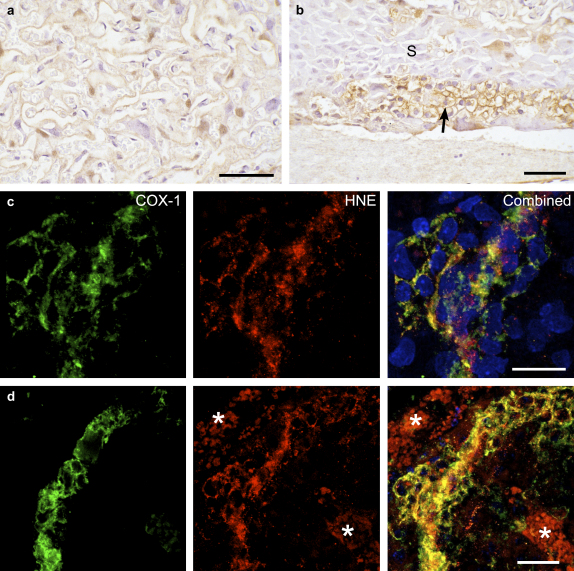
Immunoreactivity of COX-1 at E18. IHC demonstrating a) strong immunoreactivity in the syncytiotrophoblast and some cytotrophoblast cells of the LZ; and b) the GCs (arrowed) in the JZ. The spongiotrophoblast cells (S) are notably weakly stained. Fluorescent double-labelling for COX-1 and HNE showed strong co-localisation in the trophoblast of the LZ (c); and a band of GCs in the JZ (d). Note autofluorescence (*) in maternal erythrocytes in the blood spaces. Blue, DAPI. Scale bars: 50 μm.

At E14 COX-1 immunoreactivity was not observed in any cell type, except for low intensity (level 1) staining in the GCs. At E16 strong reactivity (3) was visible in the GCs, with lower intensity staining (1) being present in the spongiotrophoblast and cytotrophoblast cells (data not shown). By E18 there was a general increase in staining, with many of the cytotrophoblast cells (2) reacting positively, along with the syncytiotrophoblast (2), and all GCs (3) (Fig. 2a,b). This pattern persisted at E19, when the decidua (2) was also immunoreactive. By contrast, the spongiotrophoblast cells displayed low (0.5) immunoreactivity throughout gestation.

Towards term COX-1 and HNE immunoreactivity were present within the same cells in both the LZ and JZ, as evidenced by the dual-labelling experiments (Fig. 2c,d).

### 3.3 COX-2

Western blotting revealed a significant change in concentrations of COX-2 across gestation (*P* = 0.002), peaking at E19 (Fig. 1).

**Fig. 3:**
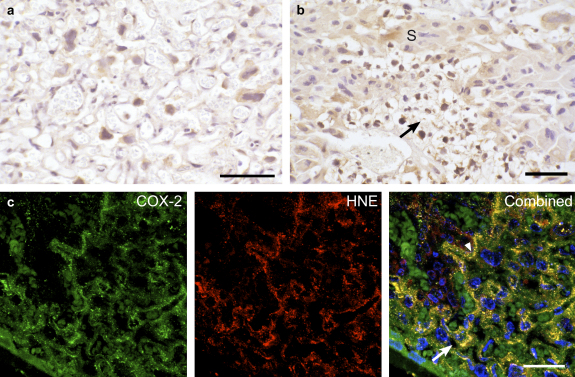
Immunoreactivity of COX-2 at E18. IHC illustrating in a) strong immunoreactivity in the cytotrophoblast cells and weaker staining in the syncytiotrophoblast of the LZ. In the JZ b) staining was particularly intense in the GCs (arrowed), and to a lesser extent in the neighbouring spongiotrophoblast (S). c) fluorescent double-labelling for COX-2 and HNE in the LZ showed strong co-localisation in cytotrophoblast cells (arrowed) but also in some stretches of syncytiotrophoblast (arrowheads). Blue, DAPI. Scale bars: 50 μm.

IHC demonstrated that COX-2 expression is low (level 1) within the syncytiotrophoblast, spongiotrophoblast and GCs at E14. With advancing gestational age immunoreactivity increased, principally in the cytotrophoblast cells (2), GCs (2.5) and spongiotrophoblast (1) at E18 and E19 (Fig. 3a,b). The syncytiotrophoblast displayed low immunoreactivity (0.5) throughout gestation.

Again there was strong co-localisation between immunoreactivity for COX-2 and HNE (Fig. 3c).

### 3.4 Detection of apoptotic changes

**Fig. 4:**
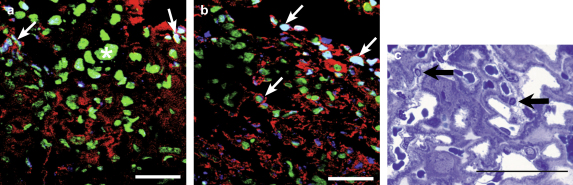
Dual-labelling co-localisation experiments for: a) TUNEL (blue) and HNE (red); and b) TUNEL (blue) and active caspase-3 (red) with DAPI (green). Most TUNEL-positive nuclei (arrows) are surrounded by cytoplasm immunoreactive for HNE or active caspase-3. By contrast, TUNEL-negative nuclei (asterisk) display no surrounding immunoreactivity. c) Resin-embedded sample of the labyrinth at E19 illustrating nuclei (arrowed) of smaller size with peripheral chromatin condensation that are considered to be undergoing apoptotic changes. Scale bars: 50 μm.

During apoptosis cytokeratins within trophoblast cells are cleaved by active-caspase 3 to yield a specific product detected by the M30 antibody [24]. The labyrinth showed low immunoreactivity for active-caspase 3 until E18 (Fig. 4b), but thereafter it increased in intensity until E19. The staining was localised to both the syncytiotrophoblast and individual cytotrophoblast cells.

Immunoreactivity for M30 also increased with gestational age, and was most abundant at E18, declining slightly at E19 (data not shown).

In order to estimate the number of apoptotic nuclear profiles TUNEL staining was performed and an apoptotic index calculated. TUNEL-positive nuclei were first observed at E16 when the apoptotic index was 2.7%, and steadily increased in frequency until E19, when the index reached 20.0% (*P* < 0.001). There was a strong temporal and spatial association between TUNEL staining and immunoreactivity for HNE (Fig. 4a) and active caspase-3 (Fig. 4b). At E16 a number of cells were caspase-positive but TUNEL-negative. Later, almost all TUNEL-positive nuclei were surrounded by cytoplasm immunoreactive for active caspase 3. TUNEL-positive nuclei were rarely observed in the absence of caspase-positivity, when they were considered to be necrotic.

To further confirm trophoblast cells were undergoing apoptosis, morphological evidence was sought by examining resin embedded samples. Cells displaying peripheral nuclear chromatin condensation, some in the classical crescent shape, were seen with increasing frequency towards E19 (Fig. 4c). There was occasional evidence of apoptotic body formation, but other classical characteristics, such as plasma membrane blebbing, could not be resolved with light microscopy. The position of the nuclei suggested that the majority was within the syncytiotrophoblast, but the involvement of cytotrophoblast and endothelial cells could not be excluded at the light microscopic level.

### 3.5 Ex-vivo culture experiments

To test the relationships between oxidative stress and the induction of COX enzymes and apoptosis experimentally, placental explants were subjected to hypoxia/reoxygenation (H/R), a known stimulus for the generation of reactive oxygen species [22].

**Fig. 5:**
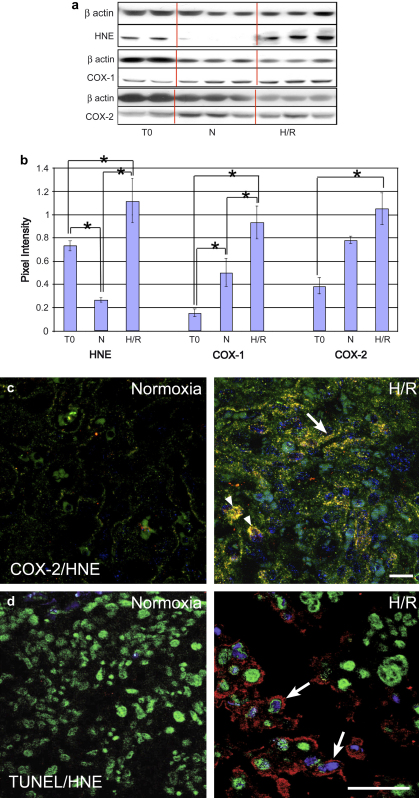
In vitro culture experiment exposing E18 placental explants to normoxia (N) or hypoxia/reoxygenation (H/R). a) Representative Western blots for HNE, COX-1 and COX-2. b) Quantification showing significant increases in each variable compared to time zero (T0) tissues. c) Dual-labelling for COX-2 (green) and HNE (red) in the LZ following normoxic culture or H/R (DAPI, blue). Following H/R there was an increase in immunoreactivity for both COX-2 and HNE which co-localised in the syncytiotrophoblast (arrow) and cytotrophoblast cells (arrowhead). d) Dual-labelling for TUNEL (blue) and HNE (red) (DAPI green) in the LZ following normoxic culture or H/R. There was an increase in TUNEL labelling following H/R, and most TUNEL-positive nuclei were surrounded by cytoplasm immunoreactive for HNE (arrow). Scale bars: 50 μm.

Western blotting confirmed increased concentrations of HNE and COX-1 in the explants following H/R compared to control samples frozen at the start of the experiment (time zero) or cultured under normoxic conditions. For COX-2 a significant increase was only observed compared to the time zero controls (Fig. 5a,b).

IHC localised the HNE to individual cytotrophoblast cells and to the syncytiotrophoblast layers of the labyrinth. In the JZ individual spongiotrophoblast cells were immunopositive, but the strongest staining was seen in the GCs within the JZ and decidua. Dual-labelling revealed strong co-localisation for COX-1 and COX-2 in the spongiotrophoblast and GCs in the JZ, and within the syncytiotrophoblast and cytotrophoblast cells in the labyrinth (Fig. 5c).

IHC revealed increased immunoreactivity for M30 within the trophoblast following H/R. The apoptotic index based on TUNEL labelling increased from 2.8% and 1.6% in the time zero and normoxic controls respectively, to 20.6% following H/R (*P* = 0.006). The majority (78%) of TUNEL positive cells were also immunoreactive for HNE on dual-labelling (Fig. 5d). From resin-embedded material it appeared that most of the cells with condensed peripheral chromatin following H/R were within the syncytiotrophoblast layers (data not shown).

From these data it can be concluded that there is a significant increase in oxidative stress following H/R, and that this is associated with increased expression of COX-1 and COX-2 expression in the LZ and JZ, and with increased apoptosis in the labyrinth.

## 4 Discussion

These results indicate that oxidative stress increases with gestational age in the murine placenta during normal pregnancies, and that it may play a significant physiological role by inducing higher concentrations of the COX-1 and COX-2 enzymes, and hence increasing prostaglandin synthesis. Our data also indicate a close temporal and spatial association between oxidative stress and trophoblast apoptosis within the labyrinth. Trophoblast apoptosis has been linked to the pathophysiology of preeclampsia, and the mouse might provide a useful genetic model in which to elucidate the mechanisms underlying the shedding of apoptotic debris.

We have recently proposed that placental oxidative stress arises through fluctuations in oxygenation [3,25], and its development in the murine placenta towards term is consistent with this view. As fetal and placental oxygen extraction reach their peaks so any transient mismatch between maternal oxygen supply and feto-placental demand will lead to a dip in the intraplacental oxygen concentration. The fact that H/R in vitro is a powerful inducer of oxidative stress, and that the stress localises to the same tissues as in late gestation supports this hypothesis. It is now accepted that oxidative stress induces a spectrum of cellular changes, ranging from the physiologically homeostatic to the frankly pathologic. Induction of enzymes is towards the more physiological end of that spectrum, whereas apoptosis is towards the opposite extreme.

Induction of the COX enzymes has been linked to oxidative stress through activation of the p38MAPK and the NF-κB family of transcription factors in other cell types [26–30]. In our study there appeared to be strong co-localisation between the formation of HNE and expression of COX-1 and -2 to individual cytotrophoblast, spongiotrophoblast and glycogen cells, both in vivo and in vitro. However, there was an apparent discrepancy between the Western blot results and our IHC findings, for the former indicated a peak in oxidative stress and COX-1 at E16 while the staining intensity for HNE and COX-1 increased until E19. This difference can be accounted for by the fact that the number of GCs declines by about half between E16 and E18 [31], so reducing their contribution to the overall tissue homogenate.

Expression of the COX enzymes appears to be mainly altered in placental rather than decidual tissues. Changes with gestational age were observed in the trophoblast populations within the LZ, but the greatest changes in immunoreactivity were seen within the GCs and the spongiotrophoblast cells in the JZ. Expression of COX-1 and COX-2 has recently been reported in the rat placenta, and that of COX-2 similarly increases with gestational age, particularly in the JZ [32]. The placental cells in this zone have direct access to maternal blood as they line the maternal venous sinuses. Therefore, it is possible that PGs synthesized in this zone in response to oxidative stress are released into the maternal circulation. It is also notable that the GCs migrating into the DB are strongly immunoreactive for COX, and so PGs synthesised by these cells could exert local paracrine effects in the decidua. As PGs have been linked with the induction of parturition [10,11,33] this raises the possibility that placental oxidative stress could play a role in initiating or augmenting uterine contractions should the mismatch in maternal blood supply and feto-placental demands become extreme.

Trophoblast apoptosis increased late in gestation as assessed by IHC for M30 and TUNEL, and cell morphology. There was a close temporal and spatial co-localisation with oxidative stress, although on Western blotting evidence of oxidative stress appeared to decline in the last days of gestation. This may again reflect changes in the cell populations within the placenta and associated decidua. The apoptotic index based on TUNEL-positivity showed a continual increase until term, although M30 immunoreactivity suggested a decline after E19. There is strong evidence that following initial cleavage, exposing the M30 epitope, cytokeratin 18 becomes further cleaved to smaller fragments [24]. In these late stages of apoptosis the M30 epitope may be lost, and so the antibody no longer detects all apoptotic trophoblast [34]. A causal link between oxidative stress and apoptosis was strengthened by the fact that apoptosis could be induced by H/R in vitro. As in the human placenta this was associated with increased concentrations of active caspase-3 [20]. The mitochondrial pathway was strongly implicated in the apoptotic process in the human, but further work is required to determine whether the same mechanism operates in the mouse.

The increase in oxidative stress and apoptosis in the LZ with gestational age may explain some of the growth dynamics of the murine placenta. Overall placental volume increases with gestational age until E16.5 and then plateaus, coinciding with the rise in oxidative stress. It is notable that within the LZ the volume and surface area of the trophoblast plateaus at E16.5 whereas those of the fetal capillaries continue to increase until E18.5 [35]. The fetal endothelial cells did not display immunoreactivity for HNE or nitrotyrosine, and did not appear to label with TUNEL, suggesting that oxidative stress and its consequences are lower in this cell type.

The links between oxidative stress and apoptosis suggest that the mouse may provide a useful genetic model in which to investigate the relative roles of antioxidant enzymes and signalling pathways in regulating trophoblast apoptosis. Apoptosis increases towards term in normal human pregnancies [36], but increased rates have been reported in preeclamptic placentas and in placentas from cases of intrauterine growth restriction [37–39]. It is thought that microparticles of apoptotic, or aponecrotic, debris released from the apical surface of the syncytiotrophoblast into the maternal circulation result in an enhanced maternal inflammatory response in preeclampsia [6]. Whether apoptotic debris is shed from the murine labyrinth as in the human placenta towards the end of gestation remains to be determined.

## Figures and Tables

**Table 1:** Antibodies and dilutions used for Western blotting and immunohistochemistry

Antigen	Supplier	Species	Clone	IHC dilution	Antigen retrieval	Western blotting dilution	Immuno-fluorescence dilution
COX-1	Cayman	Rabbit	Poly	1:250	No	1:250	
COX-1	Santa-Cruz	Goat	Poly		Yes		1:10
COX-2	Cayman	Rabbit	Poly	1:1000	Yes	1:1000	
COX-2	Santa-Cruz	Goat	Poly		Yes		1:50
HNE	Alpha Diagnostics	Rabbit	Poly	1:500	No	1:400	1:750 with retrieval
HSP 72		Rabbit				1:500	
NTyr	Chemicon	Rabbit	Poly		Yes		1:400
ß-actin	Sigma	Mouse	Mono			1:5000	
